# A surface-based cross-sectional fMRI study on brain function differences between comorbid mild or moderate depression and insomnia

**DOI:** 10.3389/fnins.2025.1554287

**Published:** 2025-05-13

**Authors:** Zhongxian Li, Limei Chen, Yingxin Huang, Luda Yan, Junquan Liang, Min Peng, Yifu Zhou, Jiliang Fang, Mengyao Li, Peng Zhou

**Affiliations:** ^1^The Seventh Clinical College of Guangzhou University of Chinese Medicine, Shenzhen, China; ^2^Shenzhen Bao’an District Traditional Chinese Medicine Hospital, Shenzhen, China; ^3^Department of Radiology, Guang'anmen Hospital, China Academy of Chinese Medical Sciences, Beijing, China

**Keywords:** surface-based study, depression, insomnia, comorbid depression and insomnia, brain function differences, functional magnetic resonance imaging (fMRI)

## Abstract

**Background:**

The mechanisms of Comorbid mild or moderate depression and insomnia (CmiDaI or CmoDaI) are complex, and stratification remains poorly understood.

**Methods:**

Resting-state fMRI data were collected from 32 patients with CmiDaI, 32 with CmoDaI, and 30 healthy controls (HCs). Data were analyzed using a surface-based computational method to examine differences in amplitude of low-frequency fluctuations (ALFF) and functional connectivity (FC) across the brain.

**Results:**

Significant ALFF differences were found in the left dorsolateral prefrontal cortex (DLPFC) between CmiDaI and CmoDaI. Compared to CmoDaI, CmiDaI showed increased ALFF in the left DLPFC, decreased FC between left DLPFC and right superior temporal gyrus, and increased FC in the right supramarginal gyrus (SMG) and right inferior frontal gyrus (IFG). Correlation analysis suggests lower left DLPFC ALFF correlated with more severe depression and insomnia. Lower FC between left DLPFC and right IFG was associated with more severe depression, while lower FC between left DLPFC and right SMG correlated with more severe insomnia.

**Conclusion:**

Our findings suggest that reduced ALFF in the left DLPFC may serve as the potential biomarker to distinguish CmiDaI from CmoDaI, and offer insights for the two disorders’ treatments.

## 1 Introduction

Depression is a common mental illness characterized by low mood, loss of interest, and lack of pleasure. Severe depression can even lead patients to commit suicide. According to the first national epidemiological survey of mental disorders in China, the lifetime prevalence of depressive disorders among adults is as high as 6.8%, and it has shown a consistent upward trend over the years (Huang et al., 2019). And the annual prevalence of major depression among US adults stood at approximately 7.0% for men and 10.4% for women (Substance Abuse and Mental Health Services Administration, 2023). Additionally, approximately one-third of adults experience insomnia, characterized by difficulty falling asleep, maintaining sleep, or early awakening (Lancet, 2022). Depression and insomnia (DAI) are often interrelated, with each condition potentially triggering or exacerbating the other. When depression is comorbid with insomnia, the symptoms are generally more severe than those of either condition alone, involving more complex pathological mechanisms and posing greater challenges for treatment. Both conditions significantly impair the quality of daily life and adversely affect patients’ overall health (Feng et al., 2019; Chinese Society of Sleep Disorders, 2023).

The etiology of depression is complex, leading to significant individual variations in symptoms. Currently, patients still show a 50–80% recurrence rate after receiving antidepressant treatment. Therefore, identifying depression subtypes in advance is essential, particularly in more complex cases involving both depression and insomnia, especially during the acute stage. This stage typically lasts about 8 to 12 weeks, with the primary focus being symptom relief and achieving the highest possible degree of clinical recovery (Simon et al., 2024). Depression exhibits significant individual variation, and its diagnosis relies solely on subjective scales and interviews, lacking specific objective diagnostic indicators. As a result, individualized treatment regimens represent the future direction of depression research (Tozzi et al., 2024). However, the brain mechanisms underlying insomnia and depression remain unclear, necessitating deeper research into the pathological mechanisms of both conditions. Studying the brain mechanisms in cases of mild depression with insomnia and moderate depression with insomnia (CmiDaI and CmoDaI) can contribute to more accurate diagnoses and the implementation of more effective treatment strategies.

Functional magnetic resonance imaging (fMRI) is a non-invasive imaging technique that can display the brain’s functional state (Chen et al., 2023; Shan et al., 2024b). Among these techniques, resting-state functional magnetic resonance imaging (rs-fMRI) allows scanning while subjects are in a resting state without any specific cognitive tasks (Shan et al., 2024a). Consequently, it has been widely used to study brain function in mental disorders on the basis of blood oxygen level-dependent (BOLD) signals. The amplitude of low-frequency fluctuation (ALFF) can indicate local brain function and identify abnormal brain regions, showed greater reproducibility compared to other fMRI localized metrics (Zou et al., 2008; Chen et al., 2018). Meanwhile, studies have shown that abnormal brain function often involves multiple regions rather than a single area. A growing number of studies have shown that the pathological brain regions associated with depression and insomnia involve interactions between brain networks. Functional connectivity (FC) analyses can reveal interactions between two brain regions, providing a more comprehensive view of brain abnormalities (Cole et al., 2022; Raij et al., 2023). FC abnormalities in orbital frontal, right inferior frontal gyrus (IFG), anterior insular, and other brain regions in patients with depression and insomnia (Liu et al., 2018; Gong et al., 2022). Given the strengths of both ALFF and FC analyses, we combined these analyses to gain a more complete understanding of brain function in the present study.

Previous fMRI studies have largely utilized computational methods based on the volumetric space to register MRI data into a three-dimensional coordinate space (Tucholka et al., 2012). However, the human brain is structured as a surface mesh, and the use of a volumetric space-based approach may introduce confounding elements, such as white matter and cerebro-spinal fluid, into MRI data outcomes. Therefore, an increasing number of studies are now using cortical-based algorithms to reconstruct the brain’s gray matter (GM) on a two-dimensional cortical surface, which is considered superior to volumetric methods in terms of brain registration, signal-to-noise ratio, and algorithm reproducibility. Quantitative studies have shown that the spatial localization of the most common version of the traditional approach is only 35% as accurate as that of the best surface-based method (Coalson et al., 2018).

Left DLPFC is a central stimulation brain region for several antidepressant physical therapies like rTMS and tDCS. In addition (Geoly et al., 2024; Woodham et al., 2024), the loss of top-down directional influence from DLPFC to limbic regions, may underlie neCmiDaItive biases in decision-making as observed in depressive states (Amemori et al., 2024). Moreover, the left DLPFC is used as a target for rTMS stimulation to effectively improve insomnia (Guo et al., 2023).

We hypothesized that surface-based brain function in the left DLPFC may serve as a potential biomarker for distinguish CmiDAI from CmoDaI. This could form the basis for diagnosing and treating depression comorbid with insomnia at varying levels of severity.

## 2 Materials and methods

### 2.1 Participants

All experiments were approved by the Clinical Research Ethics Committee of Bao’an District Traditional Chinese Medicine Hospital in Shenzhen (Ethical Approval Numbers: KY-2023-005-02), and the trial was registered with the Chinese Clinical Trial Registry Center (ChiCTR2300068054). All participants signed informed consent forms prior to participation. From January 2024 to July 2024, 64 patients with comorbid DAI were included in the study. These patients were recruited from the acupuncture department, neurology department, psychosomatic medicine outpatient clinic, and general outpatient clinics across 17 community health service centers at Bao’an District Traditional Chinese Medicine Hospital (Shenzhen, Guangdong Province, China).

Each patient experiencing a current episode of DAI was interviewed by at least two specialists. The interviews included a psychiatric examination, assessment, and diagnosis based on the Diagnostic and Statistical Manual of Mental Disorders, Fifth Edition (DSM-5). Patients were eligible for inclusion if they met DSM-5 diagnosis of both major depressive disorder (MDD) and insomnia disorder, the patients included were aged 18–65 years old, had at least a junior high school education, had the difficulty of falling asleep, maintaining sleep, or early awakening, the Hamilton Rating Scale for Depression (HAMD-17) scores between 8 and 24 and Pittsburgh Sleep Quality Index (PSQI) scores ≥ 10. In addition, for the CmiDaI group, HAMD-17 scores were between 8 and 17, and for the CmoDaI group, HAMD-17 scores were between 18 and 24.

Healthy controls (HCs) were matched to the CmiDaI and CmoDaI groups in terms of sex, age, and right-handedness. The inclusion criteria for HCs were as follows: age, 18–65 years; at least a junior high school education; both HAMD-17 and PSQI scores below 7; and no history of chronic diseases such as hypertension or heart disease. Additionally, HCs had not recently used anti-inflammatory drugs such as amoxicillin, or were using antidepressants within the last 6 weeks.

All participants were excluded if they had HAMD-17 scores > 24; showed suicidal tendencies or suicidal thoughts; were diagnosed as showing other acute or chronic inflammatory diseases; had a history of suicidal ideation, schizophrenia, bipolar disorder, or other mental disorders; reported drug abuse; or were pregnant or planning to become pregnant.

All the participants were right-handed, provided written informed consent to participate in the trial, and completed the HAMD-17, Hamilton Anxiety Rating Scale (HAM-A), PSQI, Insomnia Severity Index (ISI), and Patient Health Questionnaire (PHQ-9) tests within 3 days before the fMRI scan.

### 2.2 Data collection

Each participant underwent a single scan using a 3-Tesla Siemens Prisma MR system (MAGNETOM PRISMA, Germany) equipped with a 64-channel head coil. The participants were carefully positioned and instructed by the technician to wear earplugs. Additionally, all participants were advised to avoid falling asleep or engaging in any specific cognitive tasks during the scanning process.

HCs underwent scanning using the same sequence and parameters as those employed for patients diagnosed with comorbid depression and insomnia. The specific scanning parameters are as follows: for the 3D-T1 structural image, the repetition time (TR) was 2,530 ms, the echo time (TE) was 2.98 ms, the flip angle (FA) was 7°, and a 192-slice sagittal scan was performed with a voxel size of 1 × 1 × 1 mm. The field of view (FOV) was 256 × 256 mm, with a base resolution of 256. The total scanning time for this sequence was 5 min and 53 s. For the EPI-BOLD functional imaging, the scanning parameters included a TR of 2000 ms, TE of 30 ms, FA of 90°, and 33 layers. The layer thickness was 3.5 mm, with a layer interval of 0.7 mm. The voxel size was 3.5 × 3.5 × 3.5 mm, and the FOV was 224 × 224 mm. The base resolution was 64, and the phase resolution was 100%. The total scanning time for this sequence was 8 min and 8 s.

### 2.3 Data preprocessing

For data preprocessing and analyses, fMRIPrep 24.0.0 (Esteban et al., 2019) and XCP-D (Mehta et al., 2024). The principal preprocessing procedures were as follows.

#### 2.3.1 Anatomical preprocessing

Firstly, correction of T1-weighted image intensity non-uniformity using N4BiasFieldCorrection.And then, Skull stripping performed via ANTs 2.5.1. BrainExtraction.sh. Subsequently, segmentation of brain tissues (cerebrospinal fluid, white matter, and gray matter) executed using FSL (RRID:SCR_002823)’s fast. Brain surface reconstruction achieved with FreeSurfer 7.3.2’0.3.2urferac (default parameters). Spatial normalization to MNI152 space using ANTs.

#### 2.3.2 Functional preprocessing

Head motion correction implemented firstly through mcflirt in the Functional preprocessing part. Co-registration of the BOLD reference image to the T1-weighted image were then conducted using bbregister with six degrees of freedom. After Resampling to the fsLR template carried out by Connectome Workbench, the generation of gray ordinates files comprising 91,000 samples.

#### 2.3.3 Post-processing using XCP-D

Calculation of frame-wise displacement (Power et al., 2012). Regression of 36 nuisance regressors (employing the 36-parameter strategy). Application of band-pass filtering 0.01ping performed vian and insomnia. The specific scanning parameters are as followional homogeneity (ReHo) metrics. Finally, execution of z-score normalization and spatial smoothing with a full-width at half-maximum (FWHM) of 6.0 mm.

The peak areas with significantly difference of ALFF and ReHo values between CmiDaI and CmoDaI were identified as the seed regions to calculate Pearson correlation to the whole brain, the correlation maps of r values were transformed into z values using Fisher’s r-to-z transformation to calculate the FC.

Preprocessing workflow integrated several software packages—including Nipype, ANTs, FSL, FreeSurfer, Connectome Workbench2.0.1, and Nilearn—with all applicable transformations performed in a single interpolation step whenever possible.

### 2.4 Statistical analysis

The clinical data were analyzed using SPSS 26.0. Demographic data for the CmiDaI, CmoDaI, and HC groups were analyzed with one-way Analysis of Variance (ANOVA). Independent two-sample t tests were conducted to compare the duration of illness (in months), duration of the current depressive episode (in months), and the number of depressive episodes between the CmiDaI and CmoDaI groups.

The fMRI data statistic analysis were as follows: gender, age, and years of education were used as covariates. The ALFF, ReHo statistics were computed using PALM, with the *p*-value calculated via TFCE (Threshold-Free Cluster Enhancement) and permutation testing, performing 10,000 permutations. The significance criterion was set at p_fwe ≤ 0.05, with a cluster size greater than 200 mm^2^. Brain regions were reported using the Desikan–Killiany atlas. The ALFF and ReHo masks that survived after correction were extracted as regions of interest (ROIs) for FC analysis, which was computed using the same method as for ALFF and ReHo. *Post-hoc* analysis was used to compare the differences among CmiDaI, CmoDaI, and HC groups with Šidák correction, significance criterion was set at *p* < 0.017. To reduce the effect of head movements, the framewise displacement (FD) values should be less than 0.5 for each participants using the Power’ formula (Power et al., 2014), translation or rotation in any direction should be less than 2, Seven patients (3 CmiDaI patients and 4 CmoDaI patients) were excluded for FD>0.5, and one HC were excluded with translation>2.

To account for multiple testing, two-sided *p* values were adjusted according to the method of Benjamin/Hochberg (B/H) to control the false discovery rate (FDR). Correlation analysis were regarded as statistically significant if its corresponding B/H-adjusted p value was below 0.05, corresponding to an FDR of 5%.

## 3 Results

### 3.1 Clinical results

The CmiDaI, CmoDaI, and HC groups showed no significant differences in sex, age, or years of education (*p*>0.05). The duration of illness in patients with CmoDaI was significantly higher than that in the patients with CmiDaI (*p*<0.05), but the two groups showed no significant differences in the duration of current depressive episodes or number of depressive episodes (*p*>0.05). Additionally, the CmiDaI, CmoDaI, and HC groups showed no significant differences in the HAMA, PHQ9, ISI, and PSQI scores (*p*>0.05),as shown in Table 1.

**Table 1:** Sample characteristics.

Variable	CmiDaI	CmoDaI	HC	T/F/Z	*p*-value
Age^a^^,^^d^	31.50(26.25, 40.75)	32.50(26.25, 35)	29(25, 38)	1.144	0.564
Gender (male/female)^c^	7/22	8/20	12/17	2.155	0.340
Years of education^a^^,^^d^	16(12, 16)	16(12, 16)	16(12, 16)	0.694	0.707
Duration of illness (months)	8.621 ± 3.610	16 ± 6.526	–	−5.257	<0.001
Duration of current depressive episodes (months)^a^^,^^e^	4(3, 5)	4(3.25, 5.75)	–	−1.103	0.270
Number of depression episode^a^^,^^e^	2(1, 2)	2(2, 3)	–	−1.598	0.110
Baseline HAMD-17 score^b^	11.90 ± 2.16	20.39 ± 1.595	3.38 ± 1.720	606.485	<0.001
Baseline PHQ9 score^b^	8.55 ± 2.458	9.68 ± 2.554	1.93 ± 1.334	105.801	<0.001
Baseline PSQI score^b^	13.14 ± 1.684	13.04 ± 2.168	1.90 ± 1.263	530.345	<0.001
Baseline ISI score^b^	11.14 ± 1.597	11.57 ± 1.687	1.93 ± 1.280	363.710	<0.001
Baseline HAMA score^a^^,^^d^	7(5.25, 8.75)	12.5(11, 14.75)	2(1, 2.5)	75.938^c^	<0.001*^c^*

a Data that did not show a normal distribution were expressed as median (interquartile range).

b The remaining data were expressed as mean ± standard deviation (SD).

c Data were analyzed by the chi-square test.

d Data were analyzed by the Kruskal–Wallis test.

e Data were analyzed by the Mann–Whitney U Test. DAI, depression and insomnia; HC, healthy control; HC, healthy control; HAMD-17, Hamilton depression rating scale; PHQ9, Patient Health Questionnaire; PSQI, Pittsburgh sleep quality index; ISI, Insomnia Severity Index; HAMA, Hamilton Anxiety Rating Scale.

### 3.2 fMRI results

#### 3.2.1 Anova analysis

**Figure 1:**
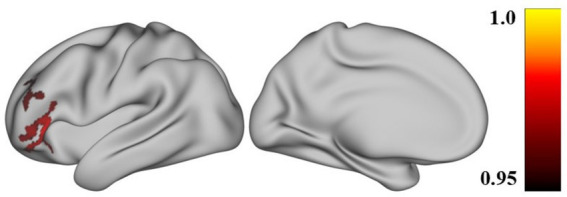
The survived ALFF in ANOVA analysis after correction. The ALFF statistics were computed using PALM, with the *p*-value calculated via TFCE (Threshold-Free Cluster Enhancement) and permutation testing, performing 10,000 permutations. The significance criterion was set at p_fwe ≤ 0.05, with a cluster size greater than 200 mm^2^.The Brain maps shows significance difference among CmiDaI group, the CmoDaI group and HC group after correction by the ANOVA analysis, and were reported using the Desikan–Killiany atlas. The figure was displayed in the view of lateral medial. The colorbar shows the mean ALFF of these clusters and their difference among every 3 groups after the ANOVA analysis. ALFF, amplitude of low-frequency fluctuation.

**Figure 2:**
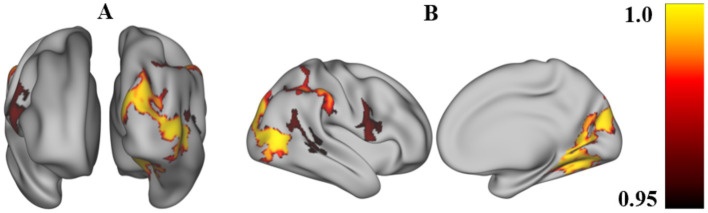
The survived FC in ANOVA analysis after correction. The FC statistics were computed using PALM, with the *p*-value calculated via TFCE (Threshold-Free Cluster Enhancement) and permutation testing, performing 10,000 permutations. The significance criterion was set at p_fwe ≤ 0.05, with a cluster size greater than 200 mm^2^. The Brain maps shows significance among CmiDaI group, the CmoDaI group and HC group after the ANOVA analysis after correction, and were reported using the Desikan–Killiany atlas. **(A)** This figure was in the view of anterior and posterior. **(B)** This figure was in the view of lateral medial. The colorbar shows the mean FC of these clusters and their difference among every 3 groups after the ANOVA analysis. FC, Functional connectivity.

1. ALFF results.The positive ALFF changes were observed in the left DLPFC and the left inferior frontal gyrus (IFG) among the three groups, as shown in Figure 1.
2. ReHo results.No significant ReHo difference results were found among the three groups (*p*>0.05).
3. FC results.The positive FC changes showed in the right inferior parietal lobule (IPL), right supramarginal gyrus (SMG), right superior temporal gyrus (STG), and right IFG, as shown in Figure 2.

#### 3.2.2 *Post-hoc* analysis

Significant differences in ALFF and FC were observed among the CmiDaI, CmoDaI, and HC group with the Šidák correction (*p*<0.017) as detailed below.

1. ALFF results.

Compared to the HC group, increased ALFF were found in the left DLPFC and left IFG in the CmiDaI group.

Compared to the CmoDaI group, increased ALFF were found in the left DLPFC in the CmiDaI group.

**Figure 3:**
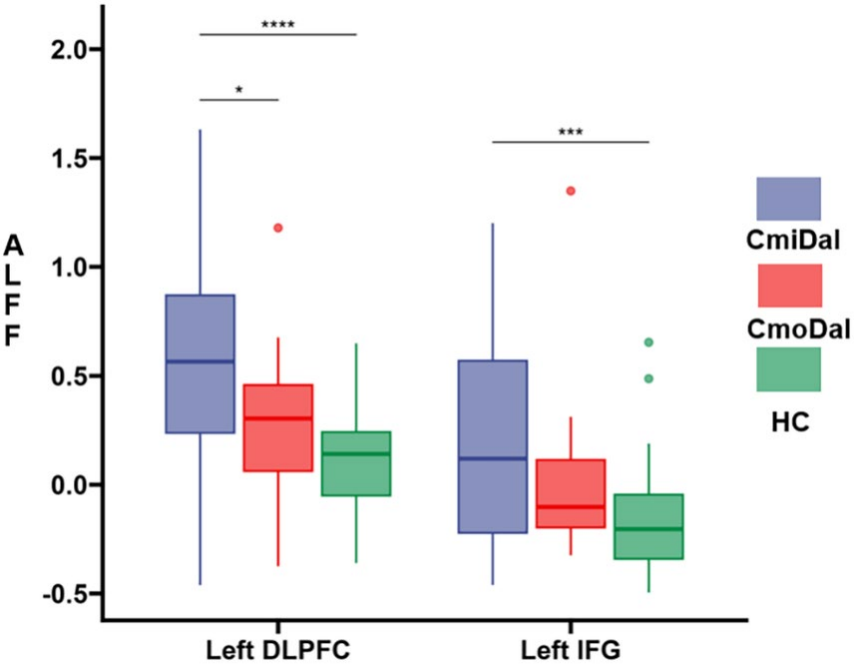
The ALFF difference among CmiDaI, CmoDaI, HC after *post-hoc* analysis. The *post-hoc* analysis performed with Šidák correction, significance criterion was set at *p* < 0.017. ALFF, amplitude of low-frequency fluctuation. DAI, depression and insomnia; HC, healthy control. DLPFC, left dorsolateral prefrontal cortex; IFG, inferior frontal gyrus. *p < 0.017, **p < 0.01, ***p < 0.001, ****p < 0.0001.

The ALFF results are shown in Table 2 and Figure 3.

1. FC results.

**Table 2:** ALFF and FC differences among the CmiDaI, CmoDaI, and HC groups.

fMRI method	Regions with FC peak	Peak coordinate	MNI coordinates (X,Y,Z)	Cluster size (mm)	*Post-hoc* analysis
ALFF	Left DLPFC	0.97	(−50,30,−1)	1,197	HC<CmiDaI,CmiDAI>CmoDaI
	Left IFG	0.96	(−44,33,22)	470	HC<CmiDaI
FC	Right IPL	0.99	(46,−66,9)	8,160	HC<CmiDaI,HC<CmoDaI
	Right SMG	0.99	(45,−40,41)	2,387	HC>CmoDaI, CmiDaI>CmoDaI
	Right STG	0.96	(54,−55,18)	1,026	HC>CmiDaI, CmiDaI<CmoDaI
	Right IFG	0.96	(57,8,18)	921	HC<CmiDaI, CmiDaI>CmoDaI

ALFF, amplitude of low-frequency fluctuation; FC, Functional connectivity. DAI, depression and insomnia; HC, healthy control. CmiDAI, mild depression and insomnia group; CmoDAI, moderate depression and insomnia group; IPL, inferior parietal lobule; SMG, supramarginal gyrus; STG, superior temporal gyrus; IFG, inferior frontal gyrus.

Compared to the HC group, the increased FC was observed in the right IPL and right IFG, while decreased FC was found in the right superior temporal gyrus in the CmiDaI group.

In the CmoDaI group, increased FC was found in the right inferior parietal lobule, while decreased FC was observed in the right SMG gyrus.

Compared to the CmoDaI group, the CmiDaI group showed decreased FC in the right STG, and the increased FC in the right SMG and right IFG.

**Figure 4:**
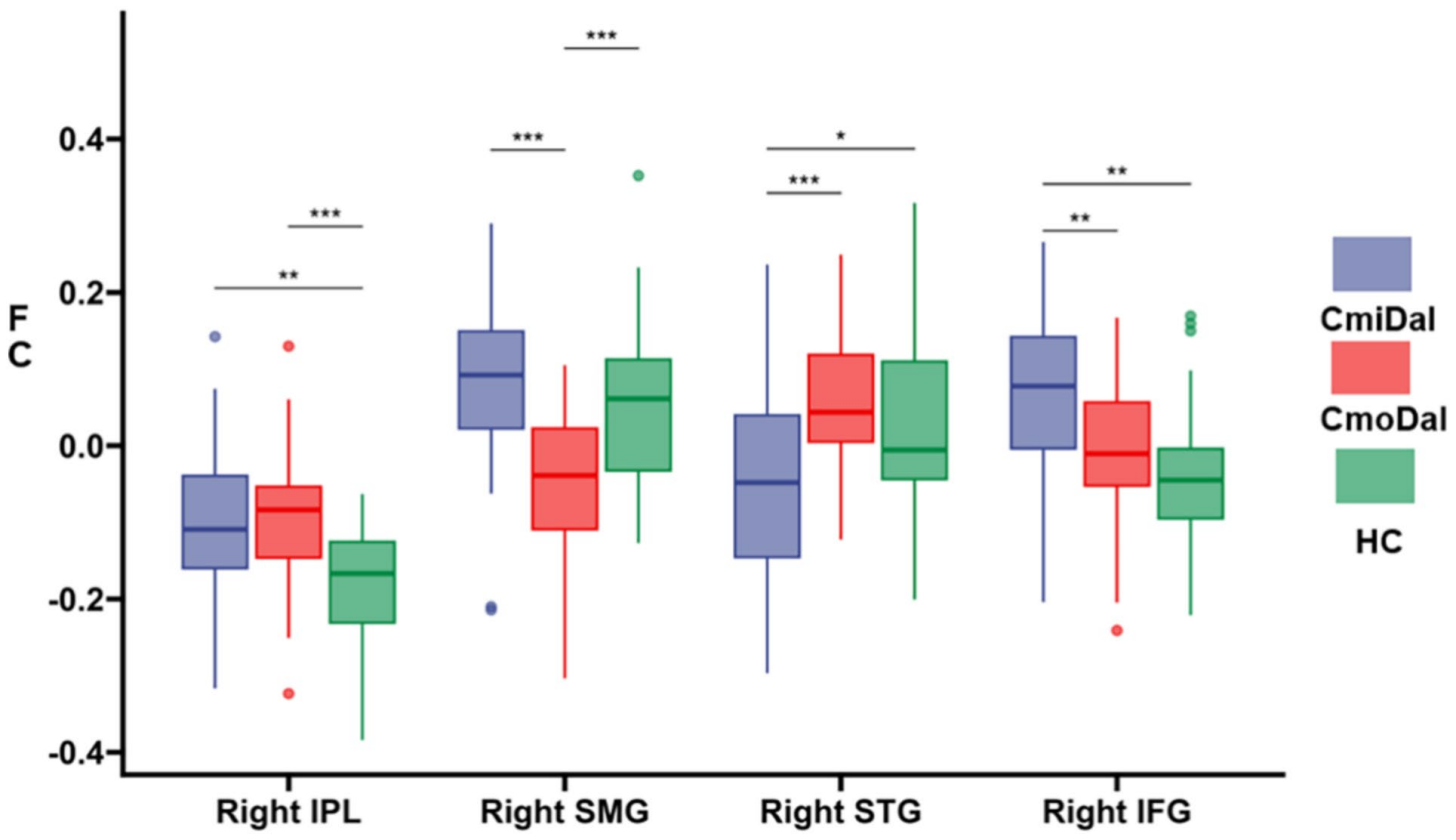
Differences in FC among the CmiDaI, CmoDaI, and HC groups after *post-hoc* analysis. The *post-hoc* analysis performed with Šidák correction, significance criterion was set at *p* < 0.017. FC, Functional connectivity; DAI, depression and insomnia; HC, healthy control. IPL, inferior parietal lobule; SMG, supramarginal gyrus; STG, superior temporal gyrus; IFG, inferior frontal gyrus. *p < 0.017, **p < 0.01, ***p < 0.001, ****p < 0.0001.

The FCs results were shown in Table 2 and Figure 4.

### 3.3 Correlation analysis

The results of correlation analysis were adjusted according to the method of Benjamin/Hochberg (B/H) to control the false discovery rate (FDR), with two-tailed *p* values < 0.05.

**Figure 5:**
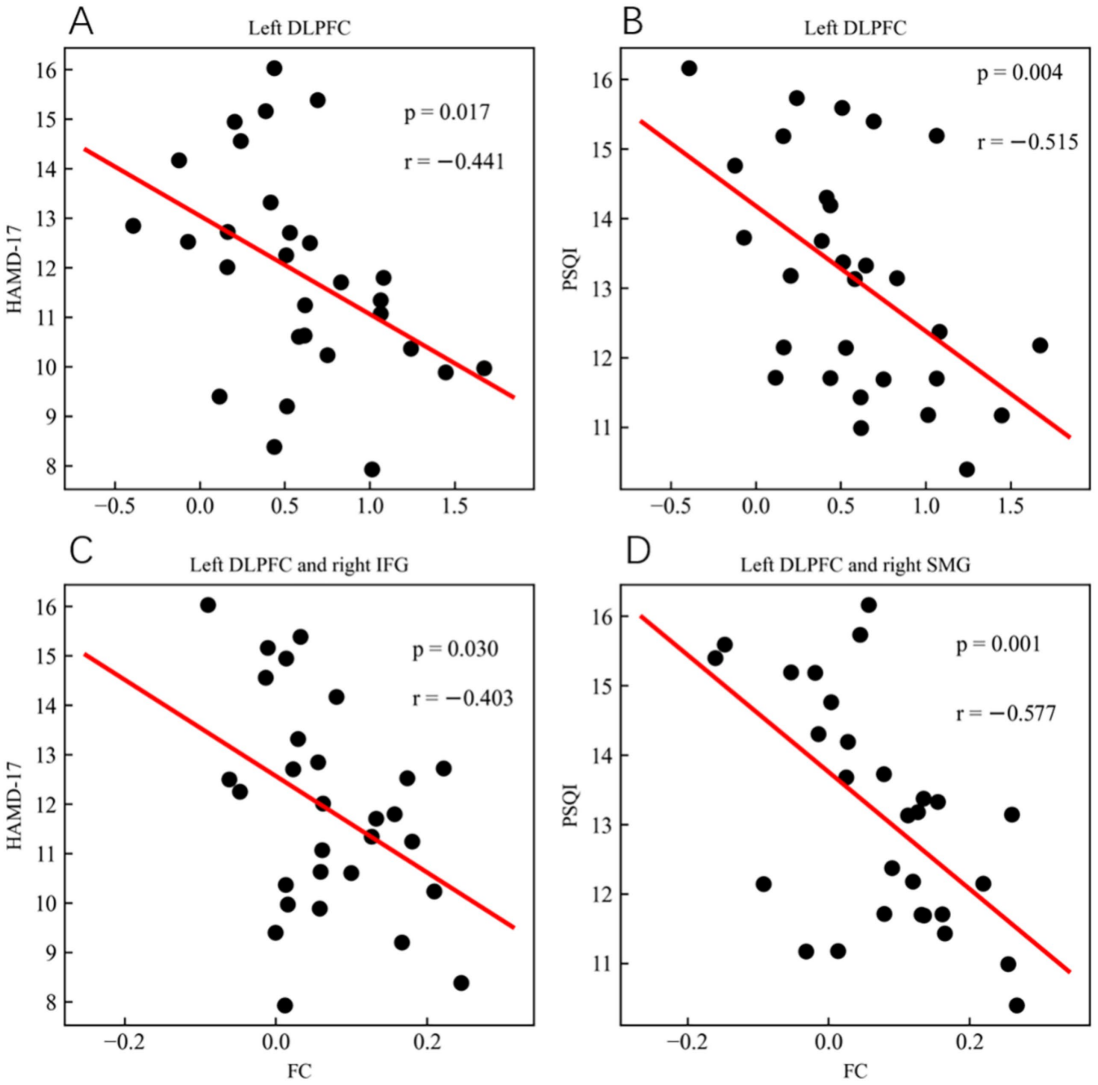
Correlations between clinical variables and resting-state fMRI. The scatter plots show significant correlation between clinical variables and resting-State fMRI. All the Clinical Variables and fMRI data were adjusted by age, gender, and education years. The CmiDAI group: **(A)** lower left DLPFC ALFF was correlated with more severe depression. **(B)** lower left DLPFC ALFF was correlated with more severe insomnia. **(C)** Lower FC between left DLPFC and right supramarginal gyrus was correlated with more severe insomnia. **(D)** Lower FC between left DLPFC and right supramarginal gyrus was correlated with more severe insomnia. ALFF, amplitude of low-frequency fluctuation; HAMD 17, 17-item Hamilton depression rating scale; DLPFC, dorsolateral prefrontal cortex; PSQI, Pittsburgh sleep quality index; FC, functional connectivity; IFG, inferior frontal gyrus; SMG, supramarginal gyrus.

1. For the CmiDAI group, lower left DLPFC ALFF was correlated with more severe depression (*p* = 0.017, *r* = −0.441; P_BH_ = 0.028; as shown in Table 2 and Figure 5A) and more severe insomnia (*p* = 0.004, *r* = −0.515; P_BH_ = 0.01; as shown in Table 2 and Figure 5B). Lower FC between left DLPFC and right inferior frontal gyrus was correlated with more severe depression (*p* = 0.030, *r* = −0.403; P_BH_ = 0.038; as shown in Table 2 and Figure 5C). Lower FC between left DLPFC and right supramarginal gyrus was correlated with more severe insomnia (*p* = 0.001, *r* = −0.577; P_BH_ = 0.005; as shown in Table 2 and Figure 5D). No significant correlation results were found between HAMA, PHQ9, ISI and fMRI results (*p*>0.05).
2. For the CmoDaI group: No significant correlation results were found between clinical variables and fMRI results (*p*>0.05).

### 3.4 Prediction of clinical symptoms

Multiple regression can predict symptoms, we using ALFF and FC as features, the results of multiple linear regression analyses indicated that the ALFF of the left DLPFC accounted for 29.2% of the variance in the mild depression group, with an IDR^2^ value of 0.292 (*p*<0.01).

## 4 Discussion

### 4.1 The left DLPFC is a potential biomarker for comorbid DAI

The prefrontal cortex (PFC) primarily consists of the dorsolateral, ventrolateral, preorbital, and ventromedial regions of the frontal cortex, of which the DLPFC projects to the dorsolateral portion of the basal ganglia (Levy, 2023). Abnormalities in the structure and function of the PFC are known to be closely related to the pathological mechanisms and treatment effects of depression (Briggs et al., 2020; Pizzagalli and Roberts, 2022). Additionally, these abnormalities are associated with key depression-related symptoms such as negative processing biases, anhedonia, and difficulties with cognitive tasks (Cools and Arnsten, 2022; Pizzagalli and Roberts, 2022). Orexin, a neuropeptide, has been shown to improve both the core symptoms of depression and insomnia simultaneously. Research indicates that orexin receptors are expressed in the PFC (Mignot et al., 2022; Chen et al., 2024), specifically the left DLPFC, which plays a crucial role in the pathology of both depression and insomnia. fMRI studies have demonstrated that CBT can improve insomnia symptoms by enhancing the activity in the DLPFC (Liu et al., 2018).

This study found increased ALFF in the left DLPFC in patients with CmiDaI. Interestingly, correlation analysis showed that a lower ALFF in the left DLPFC was associated with more severe depression scores, which aligns with our ALFF comparison results. FC analysis also revealed increased FC in CmiDaI than in CmoDaI.

Our results also showed that the ALFF of the left DLPFC can adequately distinguish CmiDaI and CmoDaI. Therefore, we used the ALFF of the left DLPFC as the seed for FC analysis. We found that the FC between the left DLPFC and the right IFG was higher in CmiDaI than in CmoDaI. Furthermore, a lower FC between these regions was correlated with more severe depression scores in patients with CmiDaI. Both ALFF and FC analyses indicated that the left DLPFC plays an important role in distinguishing between different levels of DAI.

From a clinical treatment perspective, the guidelines released by China in 2023 recommend CBT as the first-line therapy for comorbid DaI, and fMRI studies have demonstrated that CBT can alleviate insomnia symptoms by enhancing activity in the DLPFC (Jiang et al., 2024). Additionally, for the treatment of both DAI, the left DLPFC is the most commonly targeted brain region for repetitive transcranial magnetic stimulation (rTMS), which has shown promising therapeutic effects (Cole et al., 2022; Li et al., 2024; Wilckens et al., 2024).

The left DLPFC is a key node in the executive control network, which is extensively involved in tasks related to cognitive and emotional regulation (Dunlop et al., 2023). This finding indirectly supports our study’s conclusion that higher ALFF and FC in the left DLPFC are associated with milder depression. Moreover, in comparison with HCs, patients with depression exhibit a significant decrease in FC between the left DLPFC and other brain regions (Liston et al., 2014). However, study (Cheng et al., 2018) found that higher activity in the DLPFC is associated with more severe depression. The discrepancy between their findings and ours may be due to their use of big data from multiple imaging databases and the cohorts of the two studies differ in their ethnic origins, moreover, the FC results in Cheng’s study did not specify whether the increased activity was in the left or right DLPFC. Besides, the results of multiple linear regression analysis suggest that the ALFF of the left DLPFC accounted for 29.2% of the variance in the mild depression group with ID, which provides further support for our conclusion. Yet, our results showed increased ALFF in the left DLPFC in CmiDaI compared to CmoDaI and HCs. However, the correlation analysis shows that lower ALFF in the left DLPFC correlates with more severe depression and insomnia. The contradiction may be due to our sample size is relatively small, which may lead to the instability of the results (Chen et al., 2018) or lack of uniformity in patient medication regimens.

### 4.2 The important role for IFG in depression

The IFG is a key region within the PFC, which can be divided into three parts: the anterior part (pars orbitalis), the middle part (pars triangularis), and the posterior part (pars opercularis). Anatomical studies have shown that the IFG connects to the IPL via the superior longitudinal fasciculus, the IFG is crucially involved in tasks such as interoceptive awareness, emotional processing, and language as well as semantic comprehension (Briggs et al., 2019). Approximately 27% of patients with MDD exhibit significant deficits in response inhibition and cognitive impairment, which often result in poor response to standard antidepressants (Hack et al., 2023). One study (Zhuang et al., 2023) showed that the IFG is activated during response-inhibition processing, indicating its pivotal role in the pathology of depression. CmiDaI As depressive symptoms improve, the fALFF in the right IFG increases following CBT treatment, further supporting our findings showing that the FC in the IFG was higher in the CmiDaI group than in the CmoDaI group. Moreover, the FC between the left DLPFC and the right IFG in the mild depression group was neCmiDaItively correlated with HAMD-17 scores, consistent with the higher ALFF observed in the IFG in the mild depression group. Since depression episodes have been recommend to be predictive factors in the prognosis of depression (Yan et al., 2019), our study reveals the relationship between the FC left DLPFC-right IFG and the pathology and development of depression from a broader perspective. However, Patients with depression exhibited lower fractional amplitude of low-frequency fluctuation (fALFF) values in the left IFG than HCs (Schneider et al., 2020). This contrasts the findings of our study, which showed higher FC between the right DLPFC and the right IFG in the CmiDaI group in comparison with the HCs. We believe this discrepancy may be due to brain lateralization, research conducted by Brady showed that all reward-related activated brain regions were on the left side, not the right, reflecting the lateralization of depression-related frontal brain functioning (Nelson et al., 2018). In addition, the right hemisphere is more engaged in processing primary emotions such as fear, while the left hemisphere demonstrates greater proficiency in managing social emotions (Lane and Nadel, 2000). Moreover, brain heterogeneity between the left and right IFG was reported too (Güntürkün et al., 2020). Due to the relatively small sample size, no clear correlation between these changes and clinical symptoms of depression could be established in the present study.

### 4.3 SMG underlying the pathological basis for insomnia

The superior and IPL are divided by the interparietal sulcus, and SMG is one part of the IPL (Tanriover et al., 2004). The functions of the SMG include semantic and aural processing and aural execution (Oberhuber et al., 2016). Moreover, the SMG is considered to be closely linked to the pathological mechanisms underlying mental disorders (He et al., 2024). This is consistent with studies showing that acupuncture therapy can improve insomnia symptoms, and the improvement in insomnia was negatively correlated with FC in the locus coeruleus and SMG, indicating a potential mechanism by which acupuncture exerts its therapeutic effect on insomnia (Zhuang et al., 2023). These findings support the results of our study showing that the FC in the SMG was negatively correlated with PSQI.

Mechanistic research forms the foundation of clinical research, and individualization has emerged as the guiding principle and prevailing trend in the treatment of depression. Further exploration of the foundations of nuclear magnetic resonance is essential for advancing individualized treatment for depression. The recently introduced Stanford Accelerated Intelligent Neuromodulation Therapy (SAINT), which has received FDA approval for expedited treatment of TRD, has garnered significant attention due to its individualized approach based on FC assessments performed using fMRI (Cole et al., 2022).

## 5 Conclusion

In conclusion, our study provides functional brain imaging evidence of significant differences between CmiDaI and CmoDaI. These findings may enable doctors to develop more precise and effective treatment strategies for patients with depression comorbid with insomnia.

### 5.1 Limitations

Despite the significance of our findings, several limitations should be addressed. The sample size was relatively small. Larger sample sizes are needed to strengthen our findings in future studies. In addition, we need to control more tightly for differences in demographic information on depression subgroups when including subjects. Moreover, this study did not standardize the type of medication taken by the patients in both groups, and future studies need to standardize the medications taken by the patients during the inclusion of the subjects.

## Data Availability

The data that support the findings of this article are available from the corresponding author via email upon reasonable request.
